# Recommended Cardiometabolic Screening Guidelines for Unhoused Adults: A Street Medicine Needs Assessment

**DOI:** 10.3390/clinpract16040078

**Published:** 2026-04-17

**Authors:** Sanjana Arun, Joaquin Cardozo, Andre Shon Hirakawa, Teresa Anh Tran, Van Dexter Calo, Robert Fauer

**Affiliations:** 1 College of Medicine Phoenix, University of Arizona, Phoenix, AZ 85004, USA; jcardozo@arizona.edu (J.C.); andrehirakawa@arizona.edu (A.S.H.); teresatran@arizona.edu (T.A.T.); vandextercalo@arizona.edu (V.D.C.); 2 School of Medicine, Creighton University, Phoenix, AZ 85012, USA

**Keywords:** unhoused, accelerated aging, hypertension, diabetes, guidelines

## Abstract

**Background:** Unhoused individuals face disproportionately high rates of preventable chronic disease due to fragmented access to care and prolonged exposure to environmental stressors. Street medicine programs offer a mobile, low-barrier model to assess and address these unmet needs. Despite well-documented disparities, no publications in the current literature provide numerically specific screening recommendation guidelines tailored to unhoused populations. This study fills that gap using clinical data from Street Medicine Phoenix (SMP), a mobile healthcare initiative serving urban Arizona. **Methods:** We retrospectively reviewed 1322 clinical encounters recorded by SMP between August 2023 and October 2024. Diagnoses and treatments were manually categorized. Blood pressure (BP) and glucose values were analyzed using descriptive statistics and compared against national norms (CDC 50th percentile and ADA guidelines). Kruskal–Wallis and Dunn’s tests assessed age-based differences, while chi-square and Mann–Whitney U tests examined glucose patterns. **Results:** The mean patient age was 51.4 years; 34.5% identified as female. Cardiovascular issues (39.4%) and routine screenings (39.6%) were most frequently documented. Systolic and diastolic BP values were significantly elevated across all age groups except those 60+, with even the 18–39 group showing median systolic BP above CDC norms (124.0 mmHg). Among 60 patients with fasting glucose data, 41.4% met ADA criteria for diabetes, and 10.7% of those without a known diagnosis had diabetic-range values. **Conclusions:** Our findings suggest that cardiometabolic disease may emerge earlier and more aggressively among unhoused individuals than in the general U.S. population, reflecting patterns of accelerated biological aging. The elevation of cohort-based BP percentiles suggests that current national benchmarks may underrepresent clinical risk in this group. We propose initiating blood pressure screening at age 18 and fasting glucose screening by age 35 in unhoused individuals—adaptations of existing USPSTF recommendations based on cohort-specific trends. These screening thresholds can be feasibly implemented in street medicine settings to promote earlier detection and improve long-term health outcomes.

## 1. Introduction

Unhoused individuals experience an alarmingly high mortality rate compared to the general population, with one study finding a near fourfold increase [1]. Studies have consistently identified cardiovascular disease as the second-most common cause of death [1,2], with trauma [1] and drug overdose [2] as the first depending on the studied populations. Considering the growing burden of chronic health conditions within the unhoused population [3], understanding the prevalence of hypertension and diabetes within the urban unhoused population in Phoenix is an important first step in addressing this critical disparity.

Factors contributing to these disparities include limited access to healthcare, high rates of comorbid mental health conditions, substance use, and socioeconomic instability [4,5]. Cardiovascular conditions, in particular, are prevalent in this population, driven by stress, inconsistent access to medications, poor diet, and barriers to routine medical care [6]. Elevated blood pressure (BP) is a significant finding among unhoused patients, with studies demonstrating systolic and diastolic readings higher than the adult goal of 120/80 mmHg [7]. Diabetes remains underdiagnosed and undertreated among unhoused individuals, potentially leading to hyperglycemia, infections, major cardiovascular events, and subsequent hospitalization [8,9]. This indicates the need for better management of cardiovascular and metabolic health in this population.

Cardiovascular disease (CVD) represents a significant and disproportionate burden among unhoused populations. The most common conditions include heart failure, ischemic heart disease (including myocardial infarction and angina), stroke, and peripheral arterial disease. Among cardiovascular hospitalizations in homeless adults, heart failure accounts for the largest proportion (approximately 54%), followed by stroke (24%) and acute myocardial infarction (22) [8,9].

Overall, individuals experiencing homelessness have nearly a threefold higher risk of CVD compared to housed populations (pooled OR 2.96, 95% CI 2.80–3.13), with higher prevalence (11.6% vs. 6.5%) and incidence (14.7 vs. 8.1 per 1000 person-years), as well as earlier disease onset by approximately 4.6 years [8,9]. Traditional cardiometabolic risk factors are highly prevalent but often poorly controlled, with hypertension affecting up to 75.9% and diabetes 39.0% of homeless adults hospitalized for cardiovascular conditions. Despite this high burden, these conditions frequently go undiagnosed or undertreated, contributing to worse clinical outcomes in this population.

Despite the elevated risk of chronic conditions, many unhoused individuals lack regular access to primary care, leading to delayed diagnoses and inadequate management of hypertension and diabetes. This gap in care is further exacerbated by the mobility challenges of maintaining follow-up appointments and the lack of stable living conditions to support medication adherence [10]. Moreover, many street medicine initiatives focus primarily on acute issues, overlooking the chronic disease management essential for long-term health outcomes [11].

Street medicine, a healthcare model that brings medical services directly to unhoused individuals in their own environments, addresses these challenges by providing low-barrier, on-site medical care [12]. Programs like Street Medicine Phoenix (SMP) provide medical care to unhoused individuals at shelters, encampments, and public spaces across central Phoenix, Arizona. We aim to improve health outcomes through screenings, preventive measures, and targeted interventions, reducing the gap in care caused by systemic barriers [13].

This study aims to generate data-driven, field-adapted screening recommendations for cardiometabolic disease in unhoused populations, addressing a critical gap in population-specific preventive care guidelines. These insights are critical for guiding tailored healthcare strategies that effectively address the chronic disease burden within this vulnerable population.

## 2. Methods

### 2.1. Study Design and Setting

#### 2.1.1. Study Design and Setting

This is a retrospective observational study conducted through Street Medicine Phoenix (SMP), a mobile healthcare initiative affiliated with the University of Arizona College of Medicine—Phoenix. SMP delivers care to unhoused individuals at shelters, encampments, and public spaces across central Phoenix, Arizona. Street Medicine Phoenix (SMP) operates as a mobile, field-based care model in which clinicians deliver care directly to unsheltered individuals in nontraditional environments such as sidewalks, encampments, and public spaces. Encounters are typically brief (approximately 10 min per patient) and prioritize immediate clinical needs. As a result, data collection is necessarily streamlined and limited to variables that can be feasibly obtained in the field, including basic vital signs and focused clinical history. More detailed demographic, socioeconomic, and longitudinal health data are not consistently available in this setting.

#### 2.1.2. Participants and Data Collection

A total of 1322 unique clinical encounters with unhoused patients were recorded by Street Medicine Phoenix (SMP) between 12 August 2023, and 6 October 2024. Encounters were eligible if they included a legible, documented SOAP note reflecting a primary care concern (e.g., cardiovascular, metabolic, musculoskeletal, or screening-related). Notes were excluded if they lacked documentation of a clinical interaction or were illegible. An example SOAP note is provided in File S3.

Scanned SOAP notes were securely stored in UA Box Health, SMP’s HIPAA-compliant data management platform. Notes were manually reviewed by research team members to determine eligibility and extract data. Variables abstracted from each encounter included patient age, sex, systolic and diastolic blood pressure, blood glucose level (categorized as fasting, non-fasting, or unknown), known diabetes diagnosis, cardiovascular history, and treatment plan. Due to the nature of street-based care, additional variables such as weight, detailed dietary history, duration of homelessness, employment status, and ethnicity were not consistently documented and, therefore, were not included in this analysis. Routine screening encounters in this study were typically opportunistic rather than patient-initiated, occurring during outreach interactions in which clinicians offered screening based on clinical judgment and patient willingness. Additional demographic and socioeconomic variables, including ethnicity, employment status, dietary patterns, and duration of homelessness, were not consistently documented in SOAP notes and were therefore not included in this analysis. Objective measures, such as weight, weight trends, and cardiac function (e.g., echocardiographic data), were not routinely available in the field setting and were therefore not included. Specific motivations for seeking or accepting care were not consistently documented in SOAP notes. Blood pressure was measured via manual auscultation; blood glucose was obtained using fingerstick testing when clinically indicated and permitted by the patient.

All data were manually entered into a secure, de-identified spreadsheet, with secondary review by additional team members to ensure accuracy and consistency prior to analysis.

### 2.2. Data Processing and Analysis

Diagnoses and treatments were binary-coded to allow for multiple conditions and interventions per patient. Descriptive statistics summarized demographic data, diagnostic frequencies, and treatment patterns. Age and sex differences across diagnostic categories were evaluated using two-sample *t*-tests with Bonferroni correction.

To assess cardiovascular health, mean BP values were compared against standard adult targets (120/80 mmHg) and CDC 50th percentile norms from NHANES (2001–2008) [14]. Differences were assessed using two-tailed *t*-tests (α = 0.05, Bonferroni-adjusted) and visualized via dumbbell plots and heatmaps.

Fasting glucose levels (N = 60) were categorized using ADA diagnostic thresholds: normal (<100 mg/dL), prediabetes (100–125 mg/dL), and diabetes (≥126 mg/dL). Trends were visualized with histograms and bar graphs. Glucose values were further stratified by known DM status to compare diagnostic patterns, with statistical differences assessed using chi-square tests (*p* < 0.05). Skew and outliers were assessed visually.

### 2.3. Ethical Approval and Data Privacy

This study was approved by the University of Arizona Institutional Review Board (IRB) as a retrospective chart review involving de-identified clinical data. The protocol was approved under the title “STUDY00005335: Prevalence and Treatment of Primary Care Medical Issues in Unhoused Urban Patient Populations”. All methods were carried out in accordance with relevant guidelines and regulations. The University of Arizona Institutional Review Board (IRB) waived the requirement for informed consent, as this was a retrospective study involving de-identified data. No personal identifiers were included in the dataset, and all data was anonymized prior to analysis. The IRB determined that this study posed no greater than minimal risk to participants and granted a waiver of HIPAA authorization.

### 2.4. Inclusion and Exclusion Criteria

Inclusion criteria were:

- Adults aged 18 years or older;
- Unhoused individuals encountered by SMP between 12 August 2023 to 6 October 2024;
- Clinical documentation indicating a self-reported medical concern, including, but not limited, to cardiovascular, metabolic, musculoskeletal (MSK), or routine screening issues;
- Documented SOAP note containing demographic information, blood pressure, blood glucose, diagnosis, and treatment plan.

Exclusion criteria were:

- Patients under 18 years of age;
- Individuals who are housed;
- Encounters without any indication of a chief complaint or any past medical conditions;
- Incomplete or illegible notes that precluded accurate diagnostic or treatment coding;
- Non-English or non-Spanish speakers, as language barriers could compromise data integrity.

### 2.5. Diagnosis and Treatment Classification

Primary care complaints were categorized based on SOAP note documentation into clinically relevant groups, including routine screening, diabetes mellitus (DM), musculoskeletal (MSK), wound care, neurological, cardiovascular, psychiatric, respiratory, gastro-intestinal (GI), and “Other” for uncategorized issues (see File S1 for definitions). Diagnoses were binary-coded (1 = present, 0 = absent), allowing patients to be counted in multiple categories as appropriate. Cardiovascular conditions were defined based on documented complaints or self-reported history recorded in SOAP notes. This included hypertension, arrhythmias, heart failure, prior myocardial infarction, peripheral vascular disease, and symptom-based presentations such as chest pain or dyspnea suggestive of cardiac origin. These classifications were based on clinical documentation rather than confirmatory diagnostic testing. The “Other” category included a heterogeneous group of less frequently encountered conditions that did not meet thresholds for individual categorization. This category primarily included dermatologic conditions (excluding wound care), as well as genitourinary, renal, and hepatic complaints, as further detailed in the Supplementary Materials (see File S1 for definitions).

Treatment modalities were also binary-coded, allowing multiple treatments per encounter. Categories included: not addressed, wound care packages, topical creams, oral pain/fever reducers, guideline-based advice, GI medications, allergy medications/decongestants, durable medical equipment (DME), and other supportive treatments. Full definitions are provided in File S2.

### 2.6. Statistical Analysis

Descriptive statistics were used to summarize demographics, diagnoses, and treatment distributions. Continuous variables (e.g., age, BP, glucose) were reported as means with 95% confidence intervals. Categorical variables (e.g., sex, diagnostic category) were reported as proportions.

**Figure 1:**
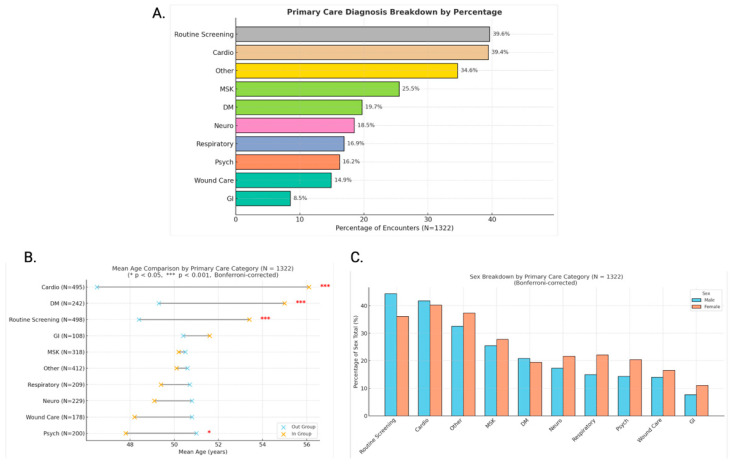
(**A**) Primary care category breakdown (N = 1322). Horizontal bar chart showing the percentage of encounters addressing each primary care issue. Routine screening (39.6%) and cardiovascular conditions (39.4%) were most frequent. Categories are not mutually exclusive; patients may appear in multiple groups. (**B**) Mean age comparison by primary care category (N = 1322). Dumbbell plot comparing mean age of patients seen for each diagnostic category (orange) versus those not seen for that issue (blue). Red asterisks denote significant differences after Bonferroni correction. N is listed for each group (* *p* < 0.05, *** *p* < 0.001). (**C**) Sex breakdown by primary care category (N = 1322). Bar chart comparing male and female representation within each diagnostic group, calculated relative to sex totals. No statistically significant sex differences were found after Bonferroni correction.

**Figure 2:**
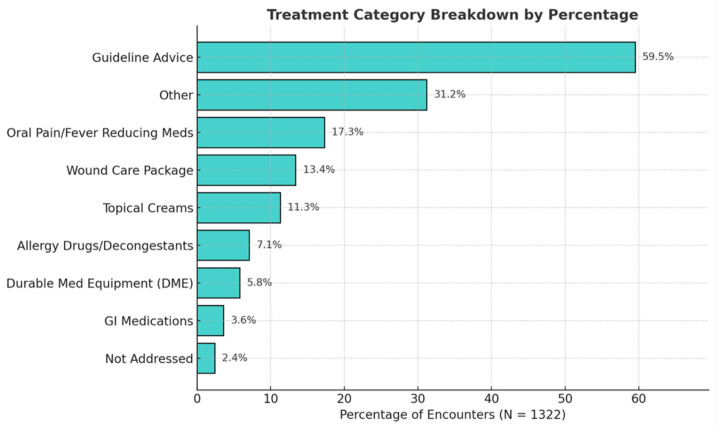
Treatment category breakdown (N = 1322). Horizontal bar chart showing treatment frequency by category. Guideline-based advice (59.5%) was most common, followed by “Other” (31.2%), pain/fever medications (17.3%), and wound care (13.4%). Multiple treatments could be recorded per encounter.

**Figure 3:**
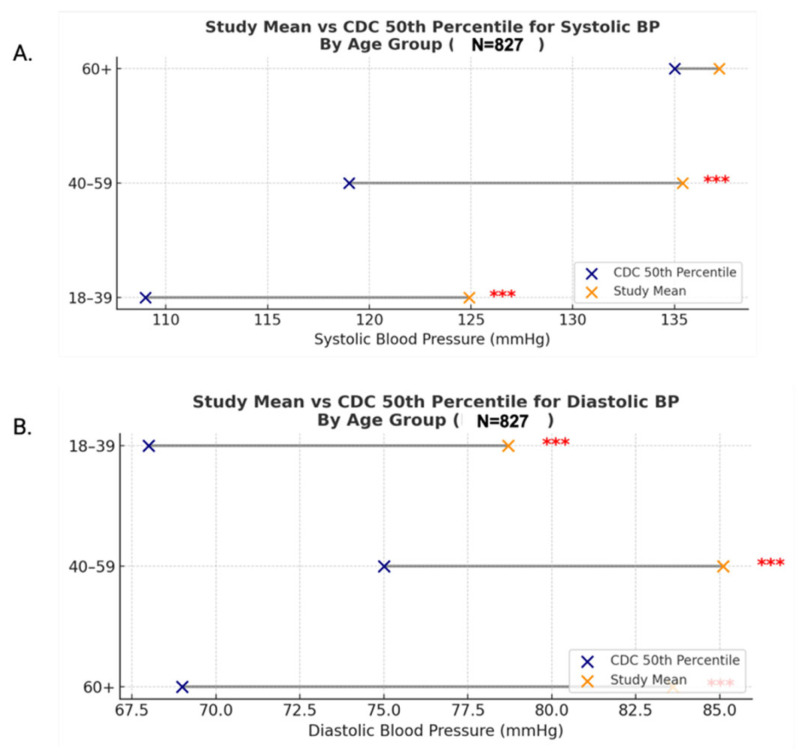
(**A**) Mean systolic BP vs. CDC 50th percentile by age group (N = 827). Dumbbell plot comparing study mean systolic BP to CDC 50th percentile norms (NHANES). Significant elevations observed in the 18–39 and 40–59 groups (*** *p* < 0.001). (**B**) Mean diastolic BP vs. CDC 50th percentile by age group (N = 827). Dumbbell plot comparing study mean diastolic BP to CDC 50th percentiles. All age groups showed significantly elevated values compared to national norms as evidenced by *** in bar graph.

**Figure 4:**
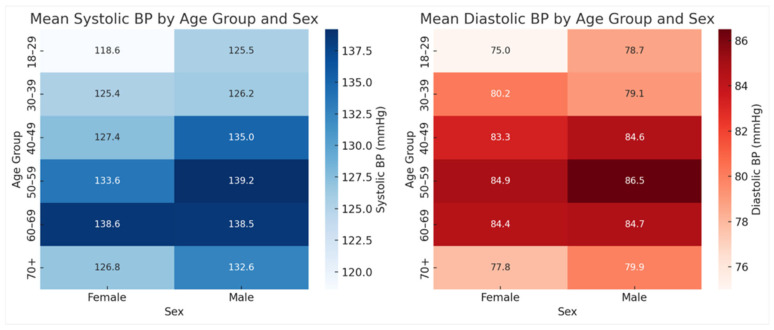
Mean BP by age Group and sex (N = 1322). Dual heatmaps of systolic and diastolic BP by age and sex. BP increased with age, with males exhibiting higher diastolic values across most age groups. Color intensity reflects pressure magnitude.

**Figure 5:**
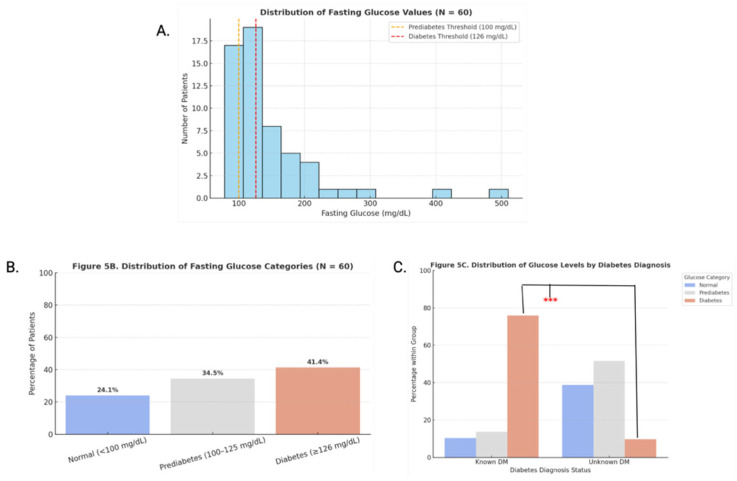
(**A**) Distribution of fasting glucose values (N = 60). Histogram of fasting glucose values with ADA thresholds marked, 100 mg/dL (prediabetes) and 126 mg/dL (diabetes). Most values clutered between 100 and 150 mg/dL. (**B**) Fasting glucose categories (N = 60). Bar chart showing proportions of normal, prediabetic, and diabetic-range glucose values per ADA criteria. A total of 34.5% were prediabetic, and 41.4% were diabetic. (**C**) Glucose distribution by diabetes diagnosis (N = 60). Bar chart comparing glucose category distribution by known diabetes status. A significant difference (as evidenced by ***) in diabetic-range values (≥126 mg/dL) was found between groups (chi-square *p* < 0.001).

- Primary care patterns (Figure 1): mean age differences across diagnostic categories were assessed using Kruskal–Wallis and Bonferroni-adjusted Dunn’s post hoc tests; sex-based differences were analyzed with chi-square tests of independence.
- Treatment patterns (Figure 2): frequencies were calculated across all modalities.
- Blood pressure analysis (Figure 3 and Figure 4): age-stratified mean BP values were compared to CDC 50th percentiles from NHANES Table 11 (2001–2008) using one-sample *t*-tests (α = 0.05, Bonferroni-adjusted) [14]. Quartiles were calculated and visualized via dumbbell plots and dual heatmaps.
- Glucose analysis (Figure 5): fasting glucose values (N = 60) were categorized per ADA criteria: normal (<100 mg/dL), prediabetes (100–125 mg/dL), diabetes (≥126 mg/dL). Group comparisons (e.g., by diagnosis status or sex) were conducted using chi-square and Mann–Whitney U tests. Visualizations included histograms and grouped bar charts.

All analyses were performed using Python (v3.10), with Pandas 3.0.1, NumPy 2.4, SciPy 1.17.1, Seaborn (https://seaborn.pydata.org/), and Matplotlib 3.10.0 for computation and visualization.

## 3. Results

From 12 August 2023 to 6 October 2024, Street Medicine Phoenix (SMP) conducted 1322 unique patient encounters, offering a valuable needs assessment of unhoused individuals in Maricopa County. Of these, 789 patients had only one documented visit, while 189 were seen multiple times during the study period. This mix of episodic and repeat engagement reflects both acute and chronic care needs in the population. The average age was 50.62 (95% CI [49.88,51.36]) with 65.37% of encounters involving males and 34.55% involving females (Table 1).

Clinical presentations were diverse, with cardiovascular issues (39.4%) and routine screenings (39.6%) emerging as the most frequently addressed primary care concerns, followed by musculoskeletal (MSK) conditions (25.5%) and diabetes mellitus (DM) (19.7%) (Figure 1A). The high prevalence of MSK concerns aligns with previous work specifically looking at orthopedic conditions within this population. MSK concerns are routinely managed with ibuprofen and durable medical equipment [15]. Other commonly documented categories included neurological conditions (18.5%), respiratory complaints (16.9%), psychological concerns (16.2%), wound care (14.9%), and gastrointestinal (GI) issues (8.5%). Any documented past medical history or chief complaints concerning these categories were included. These categories were not mutually exclusive, and many patients presented with overlapping issues, underscoring the complexity of care required in unsheltered settings.

Age significantly influenced clinical profiles. Cardiovascular and diabetes-related encounters were more common in older adults, particularly those aged 50–59 and 60–69, whereas psychiatric complaints were more prevalent among younger individuals (Figure 1B). These differences were statistically significant following Bonferroni correction (*p* < 0.001 for both Cardio and DM; *p* < 0.05 for Psych). Despite visual sex-based differences across primary care categories—such as greater cardiovascular and MSK issues in males and more frequent screenings in females—none of these reached statistical significance after correction (Figure 1C).

In terms of care delivery, treatment patterns reflected both diagnostic acuity and limitations of practicing in nontraditional, mobile settings. Guideline-based counseling and education were provided in 59.5% of all encounters, followed by oral pain/fever-reducing medications (17.3%), wound care packages (13.4%), and topical agents (11.3%) (Figure 2). Patients who were interested in screening would receive guideline-based counseling based on their blood pressure (BP) and blood glucose (BG) levels. Various acute and chronic pain conditions were often addressed with oral pain/fever-reducing medications and topical agents. Durable medical equipment (DME), such as walkers or braces, was used in 5.8% of visits, reflecting efforts to support mobility and function in unsheltered environments. No statistically significant sex-based differences were observed across treatment modalities.

The mean systolic BP was 133.7 mmHg, and the mean diastolic BP was 83.4 mmHg—both significantly exceeding the ideal adult BP goal of 120/80 mmHg (*p* < 0.001). When compared to CDC 50th percentile values from NHANES Table 11 (2001–2008), systolic blood pressure was significantly elevated in the 18–39 and 40–59 age groups (*p* < 0.001), while the 60+ group showed no statistical difference (Figure 3A, Table 2) [14]. Diastolic pressure, however, was significantly higher across all age strata (Figure 3B, Table 3). Heatmap visualization of systolic and diastolic means by age and sex demonstrated rising pressures with age and consistently higher diastolic values in male patients (Figure 4), suggesting sex- and age-specific disparities in cardiovascular risk within this unhoused population.

Fasting glucose values were analyzed for a subset of 60 patients. These values were significantly right-skewed (Figure 5A), with many exceeding diagnostic thresholds established by the American Diabetes Association [16]. Categorizing these values revealed that 24.1% of patients had normal fasting glucose levels (<100 mg/dL), 34.5% met criteria for prediabetes (100–125 mg/dL), and 41.4% were in the diabetic range (≥126 mg/dL) (Figure 5B). The high prevalence of hyperglycemia emphasizes a critical gap in accessible chronic disease screening and management.

To further explore diabetes burden, glucose categories were stratified by documented diabetes status (Figure 5C). Among patients with known DM (N = 34), 76.5% had fasting glucose in the diabetic range, significantly higher than the 10.7% among those without a diagnosis (*p* < 0.001). While this suggests suboptimal glycemic control among known diabetics, it also highlights the potential for undiagnosed disease within the broader unhoused population. These findings support the need for expanded screening, monitoring, and continuity of care in street-based medical settings.

**Figure 6:**
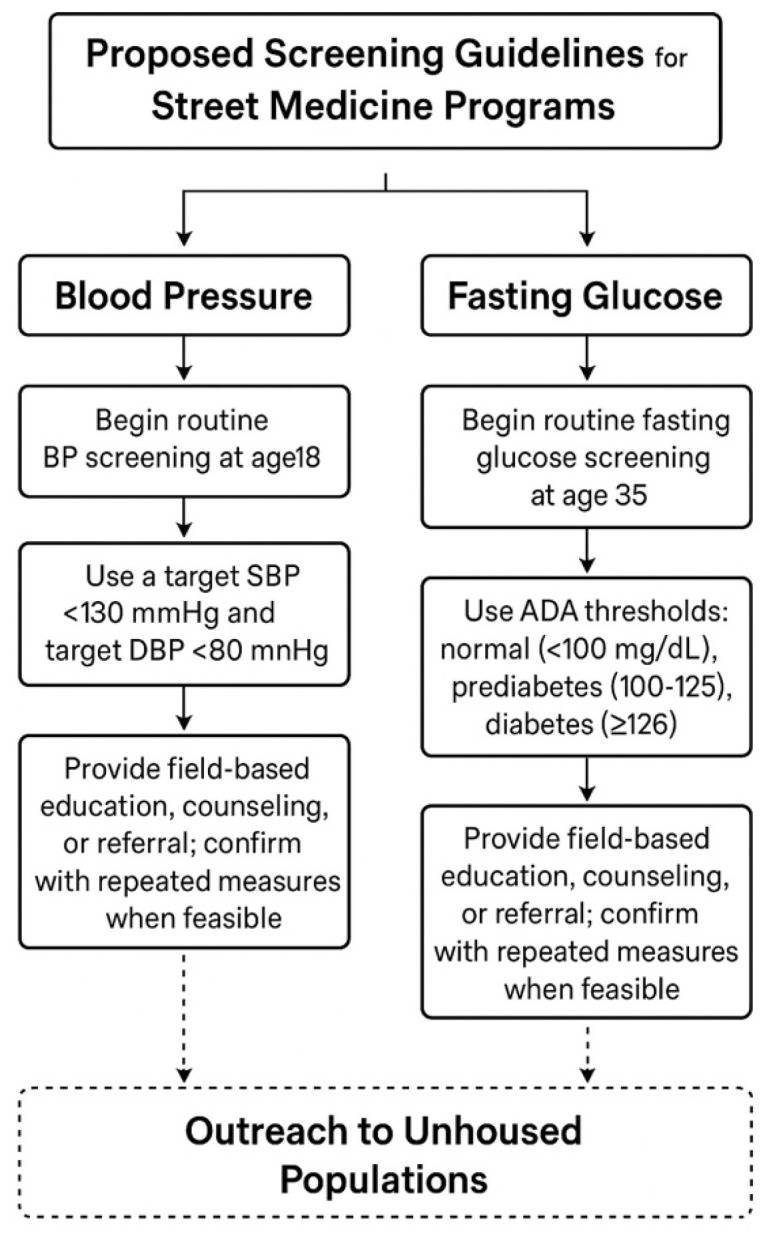
Proposed screening guidelines for street medicine. Flowchart summarizing recommended screening: BP screening beginning at age 18 (target <130/80 mmHg) and fasting glucose screening at age 35, using ADA diagnostic thresholds. Recommendations include repeat measurements when feasible and referral or counseling.

Informed by these findings, we developed a population-specific screening framework for blood pressure and glucose management in unsheltered populations, summarized in Figure 6. We propose initiating routine blood pressure screening at age 18, consistent with USPSTF recommendations, and further justified by the elevated burden of hypertension among persons experiencing homelessness (PEH), who face disproportionate rates of cardiovascular comorbidities and limited access to longitudinal care [17]. Our target thresholds—SBP <130 mmHg and DBP <80 mmHg—align with ACC/AHA guidelines and correspond to the 50th percentile values observed in our cohort [18]. For fasting glucose, 41.4% of tested patients had diabetic-range values (≥126 mg/dL), 34.5% had prediabetes (100–125 mg/dL), and 10.7% of those without a known diagnosis still met diabetic criteria. Many patients with hyperglycemia presented for unrelated concerns, emphasizing the diagnostic value of routine, opportunistic screening. These findings support a screening start age of 35 for fasting glucose, tailored to risk trends in unhoused populations. The proposed framework incorporates field-based testing, patient education, and referral strategies that are feasible in mobile care environments.

## 4. Discussion

Although a formal diagnosis of hypertension requires two or more properly measured readings according to the American College of Cardiology/American Heart Association (ACC/AHA), our findings provide compelling screening-level evidence of elevated cardiovascular risk within our population. These findings must be interpreted within the context of the street medicine care model, which differs significantly from traditional clinical settings. Data are collected during brief, episodic encounters without access to comprehensive diagnostics or longitudinal follow-up. Unlike traditional healthcare settings, preventive care in street medicine is often delivered opportunistically rather than through scheduled visits. Many individuals do not actively seek routine care, and screening is frequently initiated by the provider during outreach encounters. This model may influence both the type of conditions identified and the apparent prevalence of screening-related visits. Social determinants of health, including nutrition, employment, and duration of homelessness, likely play a significant role in the development and progression of cardiometabolic disease in this population. Future research incorporating these variables will be critical for developing more comprehensive and targeted interventions. As such, this study represents a screening-level assessment of cardiometabolic risk rather than a complete clinical or socioeconomic characterization of the population. Over 50% of participants older than 40 had at least one systolic blood pressure (SBP) reading ≥130 mmHg, corresponding to ACC/AHA Stage 1/Stage 2 hypertension (Table 2) [18]. Our reported mean SBP is also twice as high as the average reported in a sample of 97,366 people experiencing homelessness (PEH) from 1980 to 2014. [19]. In addition, whereas only 39.4% of patients in our sample reported having a cardiovascular condition, more than 50% of participants over 40 had elevated BP readings (Figure 1A, Table 2). Our data supports a lack of disease awareness in the unhoused population, potentially due to a lower rate of hypertension diagnosis [20].

Our age-compared cohort analysis based on CDC data for housed populations indicates elevated SBPs for both our 18-39 and 40-59 PEH (people experiencing homelessness) age groups, supporting both a higher cardiovascular risk in younger PEH, as well as the accelerated aging effects of homelessness (Figure 3, Table 2). Even a modest increase of 20 mmHg in SBP or 10 mmHg in DBP is associated with a twofold increase in stroke mortality [21]. This increased risk, along with our findings of elevated BP in individuals as young as 18, highlights the importance of addressing the numerous barriers to early disease monitoring and management among PEH to prevent future cardiovascular morbidity and mortality [20,22]. As shown in Table 2, systolic blood pressure in the 60+ age group was not significantly different from CDC 50th percentile norms, whereas younger age groups demonstrated significantly elevated values. This finding may reflect the high baseline prevalence of hypertension in the general older adult population, resulting in convergence between cohorts. In contrast, the elevated systolic blood pressure observed in younger unhoused individuals may suggest an earlier onset of cardiometabolic risk, potentially reflecting the cumulative effects of chronic stress and limited access to care. These findings are consistent with the concept of accelerated physiological aging in vulnerable populations. However, interpretation is limited by the smaller sample size in older age groups and the cross-sectional nature of the data.

We thus propose routine blood pressure screening beginning at age 18 in PEH based on U.S. Preventative Services Task Force (USPSTF) recommendations and the additional risk factors of low socioeconomic status, limited access to care, and high prevalence of comorbid conditions that disproportionately affect PEH [17]. Our proposed clinical targets are SBP < 130 mmHg and DBP < 80 mmHg, which align with ACC/AHA guidelines and reflect the mean of the 50th percentile of our 18–39 and 39–59 population (Table 2, Table 3) [18].

Fasting glucose screening similarly revealed widespread hyperglycemia: a total of 41.4% of tested patients had values in the diabetic range (≥126 mg/dL) and 34.5% had prediabetic levels (100–125 mg/dL) (Figure 5) [16]. Notably, among individuals without a prior diagnosis, 10.7% had glucose levels consistent with diabetes. These findings highlight the high diagnostic potential of point-of-care glucose screening in mobile medical settings—particularly for patients with limited access to routine laboratory testing or follow-up care. Moreover, a significant proportion of patients with abnormal glucose values presented with unrelated complaints, underscoring the importance of consistent opportunistic screening.

The USPSTF gives a Grade B recommendation for screening asymptomatic, overweight adults aged 35–70 for type 2 diabetes and prediabetes [23]. In its discussion of risk factors, the USPSTF emphasizes the strong link between type 2 diabetes and social determinants of health, including socioeconomic status, diet, and physical environment [23]. Given the profound impact of these factors among PEH and the challenge of weighing all patients in the street medicine setting, we recommend routine fasting glucose screening for all patients aged 35 years and older (Figure 6). Earlier screening can be considered in those with increased body habitus or comorbidities that increase their risk of developing type 2 diabetes.

Our proposed BP and BG screening guidelines reflect the physiological acceleration of aging in the unhoused population and the earlier emergence of cardiometabolic chronic diseases [20,22]. The earlier onset of hypertension and diabetes observed in this population likely reflects a combination of biological, behavioral, and social factors. Chronic stress and increased allostatic load, substance use, nutritional instability, and limited access to preventive care may all contribute to accelerated cardiometabolic risk. These factors may lead to earlier disease onset compared to the general population and underscore the importance of early screening in this group. Implementing universal BP and BG screening with validated point-of-care tools can improve the application of early lifestyle interventions and provide indications for referrals, even if formal diagnosis occurs later. Collaboration with local clinics and health systems could further facilitate continuity of care, allowing for chronic disease monitoring beyond the initial street medicine encounter [24,25]. By leveraging data from this study, street medicine programs can develop practical screening guidelines that detect disease earlier and provide a pathway for timely intervention.

While acute care remains a fundamental component of street medicine, our findings highlight a substantial burden of chronic cardiometabolic disease that may be under-recognized in outreach settings. Cardiovascular conditions and routine screening accounted for a large proportion of encounters, underscoring the need to integrate preventive and chronic disease management into street-based care models. Optimizing long-term health outcomes in this population requires a balance between addressing immediate medical needs and implementing early screening and intervention strategies.

The proposed screening algorithm may serve as a practical tool to guide opportunistic screening in street medicine settings, while also increasing awareness among healthcare providers that younger unhoused individuals may be at elevated risk for hypertension and other cardiometabolic conditions. In contrast to general population approaches, where initiation of pharmacologic therapy (e.g., ACE inhibitors or angiotensin receptor blockers) is guided by established age- and risk-based thresholds, our findings suggest that earlier recognition and consideration of intervention may be warranted in this population. In this setting, care is often delivered in an episodic manner, similar to acute care environments, where consistent follow-up may not be feasible. Future studies should focus on longitudinal care models to better assess treatment adherence, access to care, and the long-term clinical impact of such screening strategies.

Future research should evaluate the effectiveness and feasibility of implementing these screening guidelines, increasing diagnosis of chronic conditions, and improving access to medications [20]. Previous studies indicate that addressing chronic disease within mobile healthcare models remains challenging due to limited resources and irregular follow-up [26,27]. Therefore, developing prospective research designs and integrating longitudinal data collection into street medicine programs may help track patient outcomes and provide insights into the durability of treatment effects.

Our results also call for a re-evaluation of current street medicine practices. The current model of street medicine often prioritizes acute care and immediate needs, potentially overlooking chronic disease management [28,29]. Studies have shown that immediate health crises are often prioritized over long-term management of chronic conditions for unhoused populations, which may partially explain the poorly controlled hypertension and diabetes observed in our sample [30,31]. Addressing these challenges would require a paradigm shift in street medicine, integrating more preventive approaches to the care of PEH [32].

## 5. Limitations

Limitations of this study include its retrospective design, reliance on single-encounter measurements, and inability to confirm diagnoses such as hypertension, which require repeat assessments. Data were collected within a single urban setting, potentially limiting generalizability. Longitudinal follow-up, treatment adherence, and clinical outcomes could not be assessed due to the episodic nature of street medicine encounters, in which care is often delivered without the expectation of consistent follow-up. Additionally, interpretation of clinical encounters was constrained by variability in documentation and SOAP note legibility, although inter-rater review was performed to improve consistency. Blood pressure measurements may have been influenced by environmental conditions, patient factors, or provider variability despite standardized training.

Due to the field-based nature of street medicine, data collection was necessarily limited. HbA1c testing was not routinely performed, and only a subset of patients (N = 60) had fasting glucose values available. Detailed demographic and socioeconomic variables—including ethnicity, employment status, nutritional patterns, and duration of homelessness—were not consistently documented and therefore could not be included. In particular, nutritional factors, which play a critical role in cardiometabolic health, could not be systematically assessed due to the brief and opportunistic nature of encounters. Similarly, objective measures, such as weight, longitudinal weight changes, and cardiac function, were not available. Patient motivations for seeking or accepting care were not systematically recorded, as encounters were typically brief and opportunistic in nature.

Acute clinical outcomes, including real-time events such as myocardial infarction and stroke, as well as mortality, were not captured due to the episodic nature of encounters and lack of longitudinal follow-up. Patients requiring higher-acuity care were referred to external health systems, and these outcomes were not available for tracking. However, prior cardiovascular history—including myocardial infarction, arrhythmia, and heart failure—was captured when documented, as defined in our classification schema. As many patients have limited prior engagement with the healthcare system, cardiovascular conditions in this study reflect a combination of self-reported history and screening-level clinical assessment rather than confirmed acute events or longitudinal outcomes.

Finally, interpretation of age-related blood pressure trends should be approached with caution. Comparisons to population-level CDC data may reflect convergence toward already-elevated baseline values in older adults, while the disproportionately elevated blood pressure observed in younger individuals may suggest earlier onset of cardiometabolic risk in this population. These findings may also be influenced by survivor bias or differences in population composition across age groups.

## 6. Conclusions

Our study presents practical guidelines to screen for chronic diseases such as hypertension and diabetes in street medicine, emphasizing a shift from acute care to long-term management of conditions among PEH. Our findings reveal a high prevalence of elevated BP measurements within the urban Phoenix PEH community, with median systolic BP values exceeding CDC-reported population medians and the American College of Cardiology’s threshold for initiating hypertension treatment. With elevated readings observed as early as age 18, we recommend initiating routine BP screening at age 18, with a target of 130/80 mmHg. We also recommend fasting glucose screening for all individuals over 35 and for younger adults when clinically indicated. We call for increased point-of-care testing during street medicine encounters, followed by immediate counseling and referral coordination.

While this study provides a cross-sectional, screening-level assessment of cardiometabolic risk in unhoused populations, further research is needed to better capture the social, behavioral, and environmental factors that contribute to disease burden. Future studies should incorporate variables such as nutrition, substance use, duration of homelessness, and access to care, alongside longitudinal follow-up to evaluate treatment adherence and outcomes. To address these gaps, our group has initiated a prospective, IRB-approved qualitative study at the University of Arizona (Protocol: “Qualitative Perspectives of Unsheltered Patients and Their Management of Chronic Health Conditions in Urban Phoenix”), which involves follow-up interviews conducted 1–3 weeks after street medicine encounters to assess patient understanding, barriers to care, and engagement with management strategies.

## Figures and Tables

**Table 1:** Demographic characteristics of study population.

Variable	Mean/%	95% CI Lower	95% CI Upper
Age	50.62	49.88	51.36
Sex: M	65.37	62.65	68.08
Sex: F	34.55	31.83	37.27

**Table 2:** Systolic BP breakdown by age, quartile, and comparison to CDC [14].

Age Group	CDC 50th Percentile	Study Mean	25th Percentile	50th Percentile (Median)	75th Percentile	*p*-Value vs. CDC 50th Percentile
18–39	109	124.8	114.5	124.0	132.0	<0.0001
40–59	119	135.4	120.0	134.5	148.2	<0.0001
60	135	137.2	125.0	135.0	150.0	0.0897

**Table 3:** Diastolic BP breakdown by age, quartile, and comparison to CDC [14].

Age Group	CDC 50th Percentile	Study Mean	25th Percentile	50th Percentile (Median)	75th Percentile	*p*-Value vs. CDC 50th Percentile
18–39	68	78.6	70.0	80.0	85.5	<0.0001
0–59	75	85.1	78.0	84.0	92.0	<0.0001
60	69	83.6	75.5	82.0	90.0	<0.0001

## Data Availability

The data presented in this study are available on request from the corresponding authors.

## Supplementary Materials

The following supporting information can be downloaded at: https://www.mdpi.com/article/10.3390/clinpract16040078/s1, File S1: Primary Care Encounter Classification Schema; File S2: Treatment Category Definitions; File S3: Standardized Street Medicine SOAP Note Template.
