# Supplementary material for: Recommended Cardiometabolic Screening Guidelines for Unhoused Adults: A Street Medicine Needs Assessment
Source: Clin Pract. 2026 Apr 17;16(4):78. doi: 10.3390/clinpract16040078 (PMC13114903; doi:10.3390/clinpract16040078)
Supplement: Supplementary file 1 [file clinpract-16-00078-s001.zip › File S1 Primary Care Encounter Classification Schema.pdf]

Primary Care Chief Complaint Categories

- Routine Screening
  - Patient requested blood glucose check and/or blood pressure check
  - Patient had chief complaint of HTN and/or DM and underwent screening for either or both conditions
- DM
  - Patient had chief complaint relating to DM or had prior history of DM
- MSK
  - Patient had condition such as arthritis, gout, or chronic pain in the muscles, joints, or bones
  - Patient had trauma involving muscles, joints, or bones
- Wound care
  - Patient requested evaluation of skin or muscle injuries including but not limited to: abrasions, lacerations, puncture wounds, burns, blisters, animal bites, insect bites, fungal infections
- Neuro
  - Patient had complaint of headache
  - Patient had previous history of neurological condition including but not limited to: stroke, headache, migraine
- Cardio
  - Patient had complaint or past history of HTN, “fluctuating” blood pressures, “high” blood pressures
  - Patient had complaint or past history of cardiovascular conditions including but not limited to: atrial fibrillation, arrhythmia, heart failure, myocardial infarction, peripheral vascular disease
  - Patient had complaint of chest pain, dyspnea on exertion, shortness of breath attributed to cardiac cause
- Psych

Primary Care Chief Complaint Categories

    - Patient had complaint or past history of conditions including but not limited to: anxiety, depression, bipolar disease, schizophrenia
    - Patient presented with symptoms indicative of potential underlying psychiatric disorder including but not limited to: paranoia, hallucinations
    - Patient had current or past use of substance use disorders including but not limited to: alcohol, tobacco, cocaine, methamphetamines, opioid medications, marijuana
- Respiratory
    - Patient had complaint or past history of conditions affecting the respiratory system including but not limited to: allergies, upper respiratory infections, pneumonia, asthma
    - Patient had complaint of chest pain, dyspnea on exertion, shortness of breath attributed to respiratory cause
- GI
    - Patient had complaint or past history of conditions affecting the gastrointestinal tract including but not limited to: GERD, diarrhea, gastroenteritis, gallstones
- Other - non-comprehensive
    - Patient had complaint or past history of conditions affecting the skin that did not constitute as a wound care complaint including but not limited to: eczema, pruritus, rash, skin cancer
    - Patient had complaint or past history of conditions relating to the genitourinary system
    - Patient had complaint or past history of renal conditions
    - Patient had complaint or past history of hepatic conditions
