# Supplementary material for: Recommended Cardiometabolic Screening Guidelines for Unhoused Adults: A Street Medicine Needs Assessment
Source: Clin Pract. 2026 Apr 17;16(4):78. doi: 10.3390/clinpract16040078 (PMC13114903; doi:10.3390/clinpract16040078)
Supplement: Supplementary file 1 [file clinpract-16-00078-s001.zip › File S2 Treatment Category Definitions.pdf]

Treatment Categories

Not Addressed

- No treatment or counseling was provided during the encounter. This may be due to limited resources, patient departure before completion, or lack of documented follow-up plan.

Wound Care Package

- Includes field-supplied materials such as hydrogen peroxide, coban wrap, bacitracin, silver gel, or ace bandages used for wound cleaning and protection.

Topical Creams

- Includes pain- or inflammation-relief creams and ointments such as diclofenac (Voltaren), capsaicin, menthol salicylate, hydrocortisone, lidocaine, or Vicks VapoRub.

Oral Pain/Fever Reducing Medications

- Includes over-the-counter analgesics such as acetaminophen (Tylenol) and ibuprofen (Advil or other NSAIDs) provided during the encounter.

Guideline-Based Advice

- Includes verbal counseling on blood pressure or glucose management, recommended timing for follow-up screenings, referrals to primary care or emergency care, and resource navigation (e.g., shelters, clinics).

GI Medications

- Includes medications provided or recommended for gastrointestinal complaints, such as proton pump inhibitors (PPIs), H2 blockers, or antacids.

Allergy Drugs/Decongestants

- Includes antihistamines or decongestants such as cetirizine (Zyrtec), loratadine (Claritin), or fexofenadine (Allegra).

Durable Medical Equipment (DME)

- Includes physical aids such as braces, splints, crutches, boots, or walkers supplied for mobility or injury support.

Other

- Includes various non-categorized supportive treatments such as eye drops, cough drops, electrolyte powders, iron tablets, or blankets distributed based on individual needs and environmental conditions.
