# Supplementary material for: Recommended Cardiometabolic Screening Guidelines for Unhoused Adults: A Street Medicine Needs Assessment
Source: Clin Pract. 2026 Apr 17;16(4):78. doi: 10.3390/clinpract16040078 (PMC13114903; doi:10.3390/clinpract16040078)
Supplement: Supplementary file 1 [file clinpract-16-00078-s001.zip › File S3 Standardized Street Medicine SOAP Note Template.pdf]

STREET MEDICINE PHOENIX SOAP NOTE

THE UNIVERSITY OF ARIZONA
Mel & Enid Zuckerman College of Public Health
Phoenix

STREET MEDICINE PHOENIX
Caring for Phoenix's Homeless

Today's Date	Medical Student Name(s)	Preceptor
/ /		**Dr. Fauer /** ____________

PATIENT INFORMATION *(please collect as much information as you can)*

Patient Name:
*First:*___________________
*Last:*___________________
DOB: _____/______/_____
Sex: M / F / ____________
Pregnant: Y / N
LMP:___________________
Language: Eng / Span / Other:
____

Allergies: Unknown / NKDA / Other:____________________________
____

Medications:____
____
____

Medical Condition(s): kidney disease / liver disease / diabetes / HTN / heart disease / HIV / cancer / other _____________________________
____
____
____

Phone: None / Declined
____

Insurance: None / AHCCCS / Medicare / ACA / Private / Other:__________________

Location: Lodestar / GLC / Andre H / SOS / Electric Co
Other:__________________

PATIENT ENCOUNTER

SUBJECTIVE *(what is the patient complaining of?)*

____
____
____
____
____
____
____
____

ROS (*write pertinent positives/negatives*)**:** ____

OBJECTIVE *(physical exam findings,* ***minimum 3 systems****)* (□ check box if photo taken)

BP:______ / ______ BG:______ (fasting / not fasting) HR:______ HbA1c:_____ Temp:____ F/C PO2:_____

GEN: ____
CARDIAC:____
CHEST: ____
OTHER: ____
____
____
____

ASSESSMENT *(diagnoses in order of most important first)*

PLAN *(instructions given to patient)*
____
____
____
____
____
____
____

Medications given:
______________________ Instructions: ______________________
______________________ Instructions: ______________________
______________________ Instructions: ______________________

Wound care given: wound pack / bandage pack /
____

Medical equipment given: ______________________________

Resources given: _____________________________________

STREET MEDICINE PHOENIX SOAP NOTE

THE UNIVERSITY OF ARIZONA
Mel & Enid Zuckerman College of Public Health
Phoenix

STREET MEDICINE PHOENIX
Caring for Phoenix's Homeless

SMP ENCOUNTER REFERENCE SHEET *(CHECK ALL THAT APPLY)*

BLOOD GLUCOSE (BG) SCREENING

Any glucose >400, or A1C>13 recommend referral within 1 month

BG screen: Non-Diabetics, not on hypoglycemics, asymptomatic

FASTING (FBS)

- <100; normal
  recheck 1-2 years.
- FBS 100-126; pre diabetic lifestyle mgmt. Recheck 6 months.
- FBS >126; likely diabetic. Lifestyle mgmt. Recommend checking labs with A1C within 3 months.
- FBS / or NFBS>200; diabetic. Check A1C if available, if not previously done within 6 months.

NOT FASTING (NFBS)

- <100; normal
  recheck 1-2 years.
- NFBS 100-126 probably normal; recheck 1 year preferably fasting.
- NFBS >126-200 Possible pre diabetic, or normal. Recommend checking FBS within 1 year.
- FBS / or NFBS >200; diabetic. Check A1C if available, if not previously done within 6 months.

BG screen: Diabetics

- **Any glucose < 150**
  Consider the need to back off on medication. Need med info and time since last meal.
- **NFBS>300; diabetic**.
  Check A1C if available, and not done within the last 3 months. If glucose>400, or A1C>13 recommend referral within 1 month. If on medication, consider medication adjustment.
- If signs of tachypnea, dehydration, hypotension, orthostasis with glucose >400, try to adjust medication strategy. Push fluids. Consider follow up within 1-2 days (UC/ER/PCP)

BLOOD PRESSURE (BP) Screening

BP screening: Asymptomatic, no Comorbidity

- **BP <130/80**
  Check yearly if >50 or biannually if younger.
- **BP 130-139/80-89**
  Lifestyle mgmt. Recheck yearly.
- **BP 140-159/90-99**
  Recheck in both arms monthly. Lifestyle mgmt and evaluation within 6 months.
- **BP 160-179/100-109**
  Recheck in 1-2 weeks. Lifestyle mgmt, recommend evaluation within 4 months.
- **BP >179/109**
  Recheck within 2 weeks, re-evaluation within 2 months, consider dosage adjustment if on medication after rechecking B/P and confirming no concomitant stimulant drug use (e.g, Methamphetamine).

BP screening: Asymptomatic w/ Comorbidity

- **BP <130/80**
  Check yearly if >50, biannually if younger
- **BP 130-139/80-89**
  lifestyle mgmt. Recheck within 6 months.
- **BP 140-159/90-99**
  recheck b/p both arms monthly. Lifestyle mgmt. and evaluation within 3 months.
- **BP 160-179/100-109**
  recheck b/p in 1-2 weeks. Lifestyle mgmt, recommend evaluation within 2 months.
- **BP >179/109**
  Recheck within 2 weeks, re-evaluation within 1 month.

BP or Diabetic Sx Symptoms

BP screening: SYMPTOMATIC, no comorbidity

- **Stroke or unstable angina**; 325 mg ASA if not allergic. Call 911
- **Stable angina**, 81 mg ASA if appropriate, recheck B/P in 1-2 weeks, consider dosage adjustment if on medication.
- **TIA resolved <24hrs**. 325 ASA if appropriate, refer in 1-2 weeks, ER if recurrent TIA/CVA
- **TIA resolved >24 hrs**. 81 ASA if appropriate, refer in 1-2 weeks. ER if recurrent TIA/CVA
- **Heart failure**: recheck B/P in 2-4 weeks, consider dosage adjustment if on medication.
- **Edema**; recheck B/P in 1-2 months. Consider dosage adjustment if on medication, or compression stockings.
- **Headache**: Assess headache, consider acetaminophen, NSAID if B/P <140/90. Recheck B/P in 2-8 weeks dependent on symptoms.

BP screening: SYMPTOMATIC, w/ comorbidity

- **HTN Screening Symptoms:**
  Headache, edema, dyspnea, TIA, chest pain
- **Hypertension Comorbidity may include**: DM, ASHD, CHF, renal disease, HIV, COPD, anemia, hepatic disease, lipid disorder.
- **Lifestyle modification on the street**: Limit alcohol and salt, limit/avoid stimulant drug use, try to lose 5 lbs in 6 months.
- ****If B/P > 160/100 give measuring device or recommend interim repeated B/P measurements at Pharmacy or Fire Station.***
- ****If BP measuring device for patient is needed – ask leads***
